# Conditions that foster workers’ experience and development in technological transitions

**DOI:** 10.1177/10519815251410886

**Published:** 2026-01-09

**Authors:** Marta Santos, Cláudia Pereira, Maria Antónia Cadilhe, Liliana Cunha

**Affiliations:** 1 Center for Psychology at University of Porto, Faculty of Psychology and Educational Sciences, University of Porto, Porto, Portugal

**Keywords:** technology, work analysis and design, participatory ergonomics, human factors and sustainable development

## Abstract

**Background:**

Studies highlighting the importance of considering different users, including operators, at all stages of a technology introduction project are becoming increasingly frequent. However, other research points out that operators are often only involved through reskilling initiatives to cope with the technologies introduced.

**Objective:**

In this exploratory study, conducted in a company within the Portuguese industrial sector, the authors aimed to analyse a technological transition in an inbound warehouse and to understand the challenges related to mobilising workers’ experience throughout the process.

**Methods:**

Qualitative and participatory methodologies, anchored in the analysis of work activity, were used to anticipate forms of activity that may emerge in I4.0 environments, associated with the possibilities of mobilizing experience and developing skills.

**Results:**

The results indicate that operators were only taken into account during the implementation phase and that no formal development initiatives were associated with the technology. Nevertheless, situational factors—particularly related to collective work dynamics and team leadership—were identified as contributing to the general perception that the process was successful.

**Conclusions:**

Intentionally incorporating the identified factors into future projects could support the sustainability of work situations and promote the continued development of workers’ skills in contexts of technological change.

## Introduction

### From reskilling to the possibility of mobilizing experience

Following the publication of the Ergonomic Work and Analysis and Training network's participation in the IEA (International Ergonomics Association) Congress in Toronto,^1^ some challenges were identified for future reflection. Among them was the need to “qualify the sustainability conditions and criteria of learning situations in training and at work” (p. S149), associated with technological and environmental changes that have an impact on workplaces.

Several documents^2,3,4^ outline the challenges associated with Industry 4.0 (I4.0) and point to the skills gap that threatens to delay companies’ ability to introduce new technologies into their contexts. The solution to this gap seems to involve these workers in upskilling and reskilling processes so they can be better prepared to work with new technologies. The discussion in these documents is, therefore, no longer based on the more catastrophist view that anticipated the disappearance of thousands of jobs as a result of the technological revolution, but rather on its profound change. This change will require workers to get involved in initial or continuous qualification training processes to update their qualifications. In the words of Silva,^5^ who systematizes the position of these documents, “Workers will either ‘adapt’ to technological imperatives or else they will have to face the obsolescence of their know-how, which is becoming more and more labile as the capabilities of the machine advance” (p. 47).

Therefore, the reference to learning in the context of technological transition processes is not innovative. However, as the analysis carried out by Barcellini et al.^6^ points out, in these processes, workers’ participation is often limited to training sessions, which are designed without necessarily taking into account previous experience and skills developed because they are perceived as being out of step with the new needs of interacting with technology.

Building on the assumption that “the industry of the future must be built around people and their know-how”,^7^^, p.40^ it is important to examine how workers’ knowledge and participation are considered in processes of industrial and organizational transformation. Previous studies have emphasized the value of experience-based knowledge for performing current tasks and preparing for future challenges.^8,9^

The current research paper attempts to address the following questions: (1) Should workers’ participation be limited to training activities that occur only after key decisions have already been made?; (2) To what extent might the undervaluation of contributions arising from accumulated work experience constrain both present performance and future development?

## Method

### Use-case of the study: context and participants

The study is part of a technological transition project developed by a supplier of technology and services company from the industrial sector in Portugal.

As the project is still in progress, at this stage, this study focuses on one of the use-cases that the company intends to test, corresponding to a pallet tracking system in the inbound warehouse. This use-case aims to introduce a technology that enables the reorganization of the warehouse, making it more reliable and agile, ensuring product traceability.

**Figure 1.**
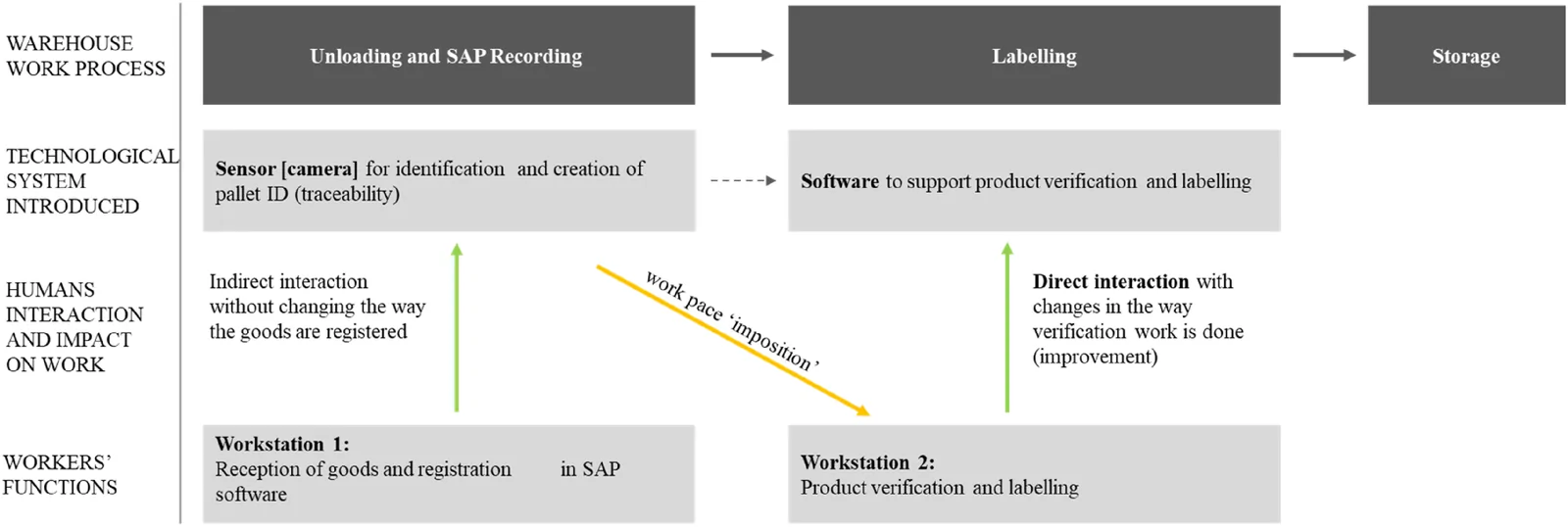
Project of digitalization of work – use-case of the study.

The inbound warehouse work process is divided into three parts: unloading goods and recording software, labelling the products, and storage. The technologies developed corresponded to integrating a sensor for identification and creating a pallet ID and, consequently, software to support product verification and labelling. Two workstations interact differently with these technologies (Figure 1). Workstation 2 (product verification and labelling) directly interacts with the software and the work pace's imposition derived from the sensor's registration of the palette at Workstation 1.

In this study participated 10 Logistics operators, ages between 26 and 57 years old (M = 40) and a seniority between 1 and 26 years old; 1 Team Leader (TL); 1 Project Manager (PM); and 1 Human Resources (HR) team member.

### Procedure

This research is an exploratory case study,^10^ seeking an in-depth understanding of a project to digitalize work in an inbound warehouse.

Qualitative and participatory methodologies, anchored in the analysis of work activity, were used to anticipate forms of activity that may emerge in I4.0 environments,^11^ associated with the possibilities of mobilizing experience and developing skills.

#### Data collection

Data collection corresponded to the analysis of micro demographic data and documents relating to the function and process of introducing the technological system (TS); Three meetings with PM (one hour each) and TL and one with an HR team member (one hour); three days of observations of Logistics operators’ work activity (logistics warehouse); and the restitution of data to workers and stakeholders (during shifts).

#### Data analysis

Data analysis consisted of the descriptive analysis of micro-demographic data, the production of tables/schemes summarizing the observations, the synthesis of the verbalizations collected, and a systematization of the data using the Enabling Collaborative Situation (ECS) framework.^12^

The results obtained focused on the analysis of those involved during the process of introducing the new technology into the warehouse, the current work situation of the operators at workstation 2, and their training and learning processes.

## Results

### Management of the process of change through the introduction of new technology

The digital transition process is formally led by the company's digitalisation team, with active involvement of the warehouse team leader in this specific use-case. The HR department recognises the importance of the ongoing transformation and the necessity of defining a strategy to manage this change, yet remains uninvolved in both the design and implementation phases of the process.

From the reconstitution of the technology implementation process in the meetings with the PM and TL, it emerged that the operators were only involved in the implementation phase of the first version of the technology (May 2022), being absent from the decision-making and design periods (beginning in 2018).

The role of the TL in the design and implementation phases stands out for his knowledge of the operators’ activities and close involvement with them.

The absence of an assessment regarding the future usefulness of the technology and the monitoring of the implementation process was effectively circumvented by the TL's in-depth knowledge of the warehouse work activity and the proximity acknowledged by the operators: “my people are not even aware of the skills they demonstrate when interacting with the system” (TL).

This became evident in the skills required and mobilised by the workers at both workstations throughout the digitalisation process. As an example, the following were validated: (i) technical skills related to the recognition of goods (e.g., the variety of suppliers and carriers requiring in-depth knowledge of the specific characteristics associated with the identifying documents of goods and products, and the different ways of handling the software accordingly); (ii) cognitive skills: analytical thinking, problem-solving associated particularly with how they must deal with the variability of supplier documentation; (iii) engagement skills such as service and customer orientation, for instance, in the care taken in handling received products and responding to production urgencies; (iv) managerial skills: quality control, ensuring a “double check” in ambiguous cases; and (iv) technological skills: digital literacy associated with mastering the software for verifying products and its interaction with the scanner and label printer.

In parallel, a production increase of around 25% was recognised, along with a reduction in errors in raw material identification: “the value does not lie in the sensor added to the process, but in the organisation of the work [time saved]” (TL).

### Analysis of the work situation according to the ECS framework

As outlined earlier, operators stationed at Workstation 2 engage directly with the software implemented to facilitate product verification and labelling. More precisely, they cross-check the type and quantity of goods received against the purchase order and generate a label which allows traceability through the scanning of barcodes and QR codes.

In the opinion of the participants, the integration of this software does not jeopardize the continuity of these operators’ work (“*The activity continues to exist*!” (TL). In fact, they feel valued that the company's project started in the warehouse and recognize how useful it is in their day-to-day work: “*We have large quantities of different products here, and the software has helped. It's wonderful because all the information is already in the QRcode*” (Log. Op. 2).

This aligns with the fact that the project was demand-driven in nature, designed to address needs explicitly identified by warehouse operators and supervisory staff: “typically, projects originate from top management. There was only one project that did not follow that pattern, and it was this one” (PM).

In addition, the analysis of the work situation through the lens of the three ECS criteria^12^ allowed for the identification of both enabling factors and constraints linked to the introduction of the new technology.

Concerning the first ECS criterion^12^ - *learning a new and more efficient way of doing things* - the operators recognize that working with this new software allows them to get the job done faster and with fewer errors. However, they identify moments when the software is unstable: “Work goes faster if [the software] is working well. Otherwise, it delays more than it helps” (Log. Op. 3). This instability leads operators to keep redundant operating modes (e.g., they keep the practice of printing out the sheets with the codes and quantities for the products they have received and need to check), which they have from previous work experience, to make sure that they don't miss any critical information or make any mistakes.

Regarding the second ECS criterion - *to increase the available possibilities and ways of doing things* -, quality instructions guiding the workflow were identified. However, operators have the autonomy to introduce variations to speed up the work process, with room to manage non-conforming situations. The operators feel that their activity is not hindered by technology and is supported by the leadership: “The guideline is to leave it alone when a [purchase order] has no data, but what we do is call the buyer or leave a message” (Log. Op. 4).

In the third criterion - *adjust the human-machine couple attributes according to the evolution of situations over time* - in addition to the possibilities of autonomy to get the job done, the operators have the opportunity to participate in improving the system: they make suggestions which are analysed and implemented according to their expertise (“The inputs [for improvement] were always from the operators” (TL); “The operation always has to be part of [the implementation]” (TL)). The autonomy granted to the operators comes from the management's recognition of their experience and ability to solve problems, particularly those related to improving technology: “When the ideas are from the operation, they stand out a lot” (TL). The operators perceive the work pace as the result of the quantity of products they receive from the pallet registration at Workstation 1 and not as an imposition by the software. However, there is a need to slow down to do a job well done when the software is working well because the pace of digital processing is slower than that of the worker.

### Training and learning issues in the analysis of the work situation

In addition to the contributions gathered from the ECS framework,^12^ the study made it possible to highlight issues from the point of view of the conditions created for the learning and training of operators in the technological transition.

It was clear from the start that there were no formal training moments for using the new software. Still, the learning took place between colleagues, according to the needs they felt, and with the support of TL: “I learned from my colleagues” (Log. Op. 2); “He [TL] came to work with the team to explain how it was done. He had a night with the team on shift” (Log. Op. 1). The learning processes were initially centred on supporting the execution of tasks deemed “routine” through the use of the new tools, with the aim of subsequently identifying strategies to address specific situations that introduced variability. During these phases, the team leader actively encouraged the reporting of problems, difficulties, or proposals for improvement.

In fact, the company's choice seems to favour a close relationship between leadership and operations since the training is designed by a department associated with the plant's operations and not the people management department.

It was also found that in the current situation, the activity is perceived as a learning opportunity due to (i) the possibility given to workers to manage problems when interacting with the system: “Everything helps us to grow. If everything is easy we don't have any difficulties, but if there are deviations from the process it will help us grow” (Log. Op. 4); (ii) the perception that this is a variable and challenging job, which requires new and adapted responses: “you never do the same thing here and that's why I like this job” (Log. Op. 2).

This would not be possible without the mobilization of previous individual experience and the use of an experienced work collective, visible in the recognition of the specificities of suppliers and purchase orders that determine the way of interacting with the software or the different products that require different handling and determine the forms of spatial and temporal organization in the interaction with the software.

## Discussion

### Challenges and criteria for the development of the activity and workers

The analysis of this use-case allows for identifying some of the challenges that, although not new, make it possible to understand the difficulties workers face in technological transition projects and which can condition the ‘use’ of their know-how and experience throughout the transition process.

Such challenges are often associated with the absence or limited involvement of workers during the design and implementation stages of new technologies, which tends to result in non-participatory and non-collaborative transitions. In these situations, system performance may be negatively affected, as well as workers’ well-being,^13^ since the transition frequently fails to address their actual needs for performing quality work.^14^ Moreover, these systems may pose risks to workers’ health, safety, and professional development, and may ultimately compromise their day-to-day activities.^15^

At the same time, in this use-case, it was possible to identify the moment when operators were involved in the transition project. As pointed out in other studies,^6^ the operators were only considered when they needed to interact with the system to produce. In other words, they were part of the actual use of the technology. One possible consequence of this late introduction of the operators may have been the inability to find a technological alternative to the need felt by the operators at workstation 2 to slow down their work pace to keep up with the machine.

Alongside this challenge, the absence of a formal training and skills development strategy for operators to develop new ways of carrying out the activity was also identified. The project managers did not foresee the upskilling of these operators, contrary to what is widely believed.^2,3,4^ Although learning took place with colleagues and TLs, there was no planning or intervention on the part of the design team to anticipate any difficulties the operators might have or to develop skills for using the system.

### Sustainability of learning situations in training and at work

Despite this, the overall positive perception of the introduction of the new system seems to be due to some conditions that reveal themselves as ways to take into account the know-how of workers in technological transition processes and that can contribute to the sustainability of learning situations in training and at work:^1^

- possibility of mobilising and further developing existing knowledge and competencies, by drawing on situated work experience, particularly given the continuity preserved with previous work practices;
- autonomy to elaborate and select the most suitable strategy for managing not only the received products but also the tools used for their identification and labelling, in accordance with the situational demands;
- workers’ perception that the introduced software is functional and supports the emergence of new ways of working, while acknowledging the dynamic nature of evolving work situations;
- perceived control over the technological system, contributing to the feeling that their work activity is supported rather than constrained;
- recognition by the team leader of the situated activity carried out by operators in their interactions with the technology and in regulating unforeseen issues from their work in the warehouse;
- opportunity to propose improvements to the system and engage in its collective use, fostering the co-development of shared operational competencies.

While these aspects are considered, as by other authors, to be essential for the sustainability of the work,^8,16^ in this use-case they are the result of a favourable, but casuistic, combination of factors. However, in order to support this sustainability, it would be important that these conditions were intentionally considered and embedded within the project from the outset.

The operators’ contribution to the success of this use-case is clear, even though they were only integrated during the use phase. As pointed out by Sgarbossa et al.,^17^ the underperformance of some aspects of the new system could have been minimized if the operators had participated in the different decision-making moments.

## Conclusions

This study aimed to analyse a technological transition in an inbound warehouse and to understand the challenges associated with mobilising workers’ experience throughout the process.

The results illustrated that, despite constraints related to the lack of worker participation in decision-making moments, there are elements that contribute to the potential for continued learning and competence development, supporting the sustainability of learning situations in both training and work, even in contexts of technological transition.

While this research focused on a single case within one company, its implications extend beyond the immediate context. The convergence between the findings and prior research, together with the similarity of organisational structures and digitalisation approaches across companies, reinforces the broader relevance of this study for analysing learning and competence development in technological transitions.

The relevance of this study lies in the identification enabling factors that can support decision-makers in understanding what sustains the mobilisation of prior experience and workers’ active participation. These dimensions should be formally, intentionally, and structurally integrated into projects and tasks associated with the introduction of new technologies.

Thus, this contribution holds significant practical implications, particularly by emphasising the need to consider certain sustainable conditions in technological transition processes. Such conditions are essential for enabling workers to continue developing their skills, build healthy and sustainable career paths, and perform quality and meaningful work. The future of work should also account for the constraints faced by digitalisation project teams and company decision-makers, promoting conditions that allow workers’ perspectives, training, and experience to be considered from the earliest stages of technological decision-making.
